# Bee Venom Phospholipase A2 Ameliorates House Dust Mite Extract Induced Atopic Dermatitis Like Skin Lesions in Mice

**DOI:** 10.3390/toxins9020068

**Published:** 2017-02-18

**Authors:** Kyung-Hwa Jung, Hyunjung Baek, Manho Kang, Namsik Kim, Seung Young Lee, Hyunsu Bae

**Affiliations:** 1 Department of Science in Korean Medicine, Kyung Hee University, 26 Kyungheedae-ro, Dongdaemoon-gu, Seoul 02447, Korea; jhkh242@naver.com (K.-H.J.); bguswjd@naver.com (H.B.); serif99@hanmail.net (M.K.); leesy4096@hanmail.net (S.Y.L.); 2 Department of Clinical Korean Medicine, Graduate School, Kyung Hee Univeristy, 26 Kyungheedae-ro, Dongdaemoon-gu, Seoul 02447, Korea; zegil@naver.com

**Keywords:** atopic dermatitis, bvPLA2, IgE, mast cell, skin lesion

## Abstract

Atopic dermatitis (AD) is a biphasic inflammatory skin disease that is provoked by epidermal barrier defects, immune dysregulation, and increased skin infections. Previously, we have demonstrated that bvPLA2 evoked immune tolerance by inducing regulatory T cells (Treg), and thus alleviated Th2 dominant allergic asthma in mice. Here, we would like to determine whether treatment with bvPLA2 exacerbates the AD-like allergic inflammations induced by house dust mite extract (DFE) in a murine model. Epidermal thickness, immune cell infiltration, serum immunoglobulin, and cytokines were measured. Ear swelling, skin lesions, and the levels of total serum IgE and Th1/Th2 cytokines were elevated in DFE/DNCB-induced AD mice. Topical application of bvPLA2 elicited significant suppression of the increased AD symptoms, including ear thickness, serum IgE concentration, inflammatory cytokines, and histological changes. Furthermore, bvPLA2 treatment inhibited mast cell infiltration into the ear. On the other hand, Treg cell depletion abolished the anti-atopic effects of bvPLA2, suggesting that the effects of bvPLA2 depend on the existence of Tregs. Taken together, the results revealed that topical exposure to bvPLA2 aggravated atopic skin inflammation, suggesting that bvPLA2 might be a candidate for the treatment of AD.

## 1. Introduction

Atopic dermatitis (AD) is a common chronic and recurrent inflammatory skin disease that results from dysregulated immune responses due to excessive stimulation by external antigens [1]. The prevalence of AD has significantly increased in recent years, and it now affects approximately 1%–3% of adults and up to 20% of children [2]. The pathogenesis of AD has been attributed to alternate mechanisms that include pollutants, allergies, genetic constitution, and immunological function [3,4,5]. The clinical features of AD patients include skin erythematous plaques, eruption, and elevated serum IgE and T helper cell type 2 (Th2) cytokine levels. AD patients also exhibit epidermal hyperplasia and accumulations of mast cells and Th2 cytokines [6,7].

Many cases of AD are treated with anti-inflammatory agents that modulate Th1 and Th2 responses and the IgE concentration. Topical steroids and other immunosuppressive agents are commonly used for the treatment of moderate-to-severe AD. Novel therapeutic approaches involve components that are important for the maintenance of the epidermal barrier, including ceramide and other components of epidermal differentiation enzymes [8]. However, the incidence and relative risk of toxicity from these agents are not insignificant, particularly in children. Recently, various natural sources—such as plants, animals, microorganisms, and herbs—have received attention as alternative therapeutics in the treatment of AD due to their proven safeties and potent immunomodulatory effects.

The bee venom (BV) of *Apis mellifera* contains a variety of peptides and proteins, including melittin, phospholipase A2 (PLA2), adolapin, apamin, and mast cell degranulating (MCD) peptide [9,10,11]. BV is an anti-inflammatory drug that has been used in the treatment of pain, arthritis, Parkinson’s disease, and multiple sclerosis [12,13,14]. PLA2 is one of the major components of BV and plays central roles in a wide range of cellular responses, such as phospholipid metabolism, signal transduction, and the regulation of inflammatory and immune responses [15,16].

Our recent study demonstrated that bvPLA2 causes immune tolerance by increasing the population of CD4^+^Foxp3^+^ regulatory T cells (Tregs) in cisplatin-induced nephrotoxicity and allergic asthma models [17,18]. The protective effects of bvPLA2 were found to be related with the modulation of Tregs. Thus, we hypothesized that bvPLA2 might be a candidate anti-inflammatory agent for AD treatment. Furthermore, the production of serum markers such as total IgE and Th1/Th2 cytokines are significantly decreased in bvPLA2-treated mice compared to the DFE/DNCB-sensitized control group. In addition to the infiltration of mast cells, epidermal hyperplasia is reduced upon bvPLA2 treatment in AD mice. We also determined the role of Tregs in the bvPLA2-mediated suppression on skin lesions. The suppressive effect of bvPLA2 was abolished following Treg depletion using PC61 anti-CD25 monoclonal antibody (mAb). Finally, this study proposes that bvPLA2 is a potential novel therapeutic agent for the treatment of AD patients.

## 2. Results

### 2.1. bvPLA2 Treatment Alleviates DFE/DNCB-Induced Skin Lesions

**Figure 1:**
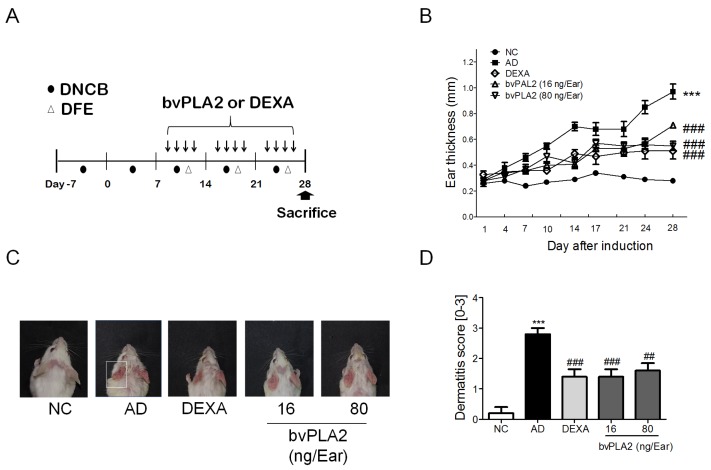
Effects of bvPLA2 treatment on DFE/DNCB-induced AD-like symptoms. (**A**) Experimental design of the study protocol. To study the effect of bvPLA2 on atopic dermatitis, mice were treated with DFE/DNCB for four weeks and bvPLA2 was injected for three weeks during antigen sensitization; (**B**) The ear thickness was measured using a dial thickness gauge 24 h after the DFE/DNCB application; (**C**) Images of the ear skin lesions of the groups of mice taken on the last day of the experiment (day 28); (**D**) The severities of the symptoms of the ear skin lesions were microscopically indexed with the dermatitis score, which was specified as the sum of the scores (0 = no symptoms; 1 = mild symptoms; 2 = moderate symptoms; and 3 = severe symptoms) for the symptoms of erythema/hemorrhage, edema, excoriation/erosion, and dryness/scaling. NC: normal control group; AD: DFE/DNCB applied group; DEXA: DFE/DNCB applied and dexamethasone-treated group (0.1 mg/Ear, 20 µL) as a positive control; bvPLA2 (16 ng/Ear): DFE/DNC applied and bvPLA2 (16 ng/Ear)-treated group; bvPLA2 (80 ng/Ear): DFE/DNC applied and bvPLA2 (80 ng/Ear)-treated group. The statistical analyses were conducted with one-way ANOVA followed by Newman-Keuls multiple comparison tests (*** *p* < 0.001 vs. NC and ^###^ *p* < 0.001, ^##^ *p* < 0.01 vs. AD; *n* = 5).

We investigated the effects of bvPLA2 on the alleviation of DFE/DNCB-induced AD-like symptoms. Mice were repeatedly subjected to chemical irritation with DNCB and antigen challenge with DFE on both ear lobes for four weeks (Figure 1A). As expected, repeated topical application of DFE/DNCB (AD group) gradually increased the ear thickness compared to normal control mice (NC group) by seven days. Treatment with bvPLA2 (16 and 80 ng/ear) and DEXA produced partially decreased ear thickness when compared to the AD group. (Figure 1B). Repeated topical applications of DFE/DNCB induced a skin swelling on the ears of the mice and elicited increased dermatitis severity as indicated by the severity of symptoms, such as erythema/hemorrhage, edema, excoriation/erosion, and dryness/scaling (Figure 1C,D). Administration of bvPLA2 showed the significant suppression of AD-like skin lesions.

### 2.2. bvPLA2 Inhibits DFE/DNCB-Induced Th1 and Th2 Cytokine Production and Serum IgE Levels

**Figure 2:**
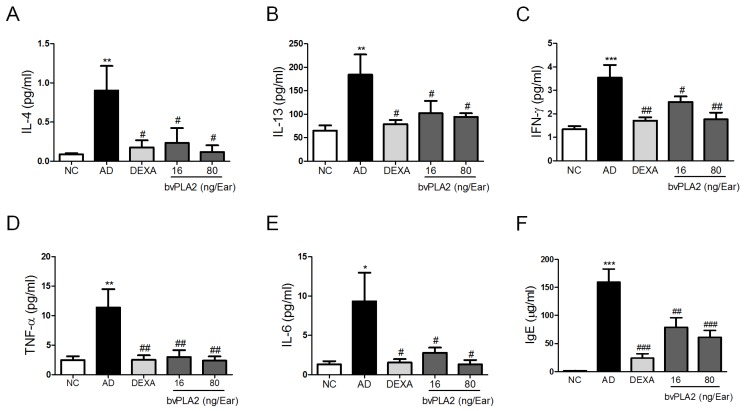
Effects of bvPLA2 on expression of cytokines and serum total IgE. The levels of (**A**) IL-4; (**B**) IL-13; (**C**) IFN-γ; (**D**) TNF-α; (**E**) IL-6 in the ear and (**F**) total IgE in the serum were quantified by mouse CBA (Th1, 2, and 17) kit and ELISA kit. The statistical analyses were conducted with a one-way ANOVA followed by Newman-Keuls multiple comparison tests (*** *p* < 0.001, ** *p* < 0.01 and * *p* < 0.05 vs. NC and ^###^ *p* < 0.001, ^##^ *p* < 0.01 and ^#^ *p* < 0.05 vs. AD; *n* = 5).

To determine the effects of bvPLA2 on DFE/DNCB-induced AD-like inflammatory responses, we measured the expression of Th1 (TNF-α, IL-6 and IFN-γ) and Th2 cytokines (IL-4 and IL-13) in the protein extracted from the ear tissues. The AD group showed significant increased levels of Th1 (Figure 2A–C) and Th2 (Figure 2D,E) cytokines, while the bvPLA2 groups exhibited significantly reduced levels of Th1/2 cytokines compared to the AD group. Furthermore, the DEXA group remarkably reduced the Th1/2 cytokine expression and resulted in a pattern of inhibition similar to that observed for the bvPLA2 (80 ng/ear) group.

Next, we examined the total IgE level in serum. Hyper-production of serum IgE is known to be a major characteristic feature of AD [19]. As shown in Figure 2F, the bvPLA2 groups showed a significant reduction of the total serum IgE level in a dose-dependent manner. As expected, the positive control (DEXA group) showed a dramatic suppression of the increase in Th1/2 cytokines as well as serum total IgE secretion. Altogether, these results strongly suggested that bvPLA2 exerted a significant inhibitory effect on Th1/2 cytokine production and hyper-production of serum total IgE in AD-like skin lesions in the DFE/DNCB-induced mice model.

### 2.3. bvPLA2 Inhibits DEF/DNCB-Induced AD-Like Histopathological Changes

**Figure 3:**
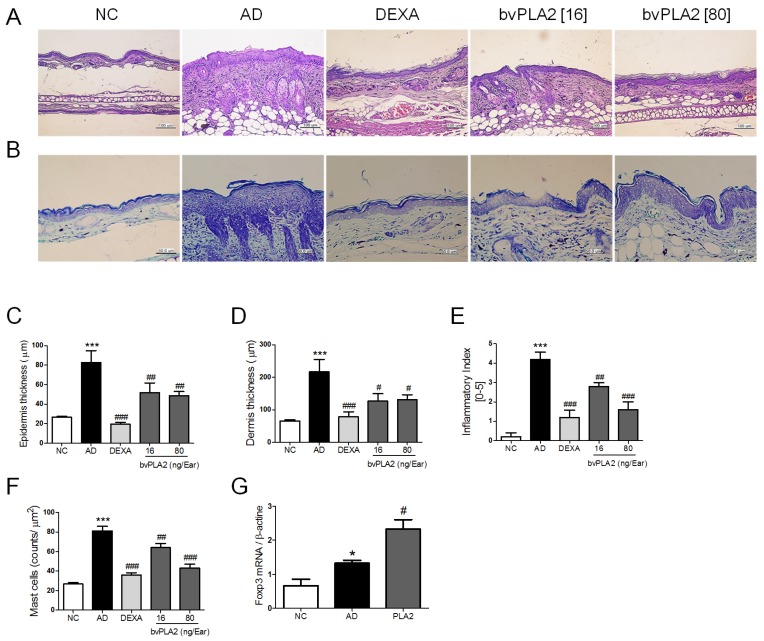
Effects of bvPLA2 on histological changes of DNCB/DFE-induced AD-like skin lesions. (**A**) The ear sections (4 µm thick) were stained with hematoxylin and eosin (H&E) and (**B**) Toluidine blue (TB) and taken with ×200 and ×400 magnification; (**C**) The thicknesses of epidermis and (**D**) dermis were quantified based on the H&E stained sections using Image Pro-Plus 5.1 software as described in the Materials and Methods; (**E**) Evaluation of ear histological change severity was quantified using a five-point score system; (**F**) The number of mast cells for five sites selected at random was counted on the ear sections with stained TB. (**G**) The expression of Foxp3 in the ear tissue was determined by real-time PCR. The statistical analyses were conducted with a one-way ANOVA followed by Newman-Keuls multiple comparisons test (*** *p* < 0.001 and * *p* < 0.05 vs. NC and ^###^ *p* < 0.001, ^##^ *p* < 0.01 and ^#^ *p* < 0.05 vs. AD; *n* = 5).

DFE/DNCB challenge caused potent histopathological changes that included thickening of the epidermis, fibrosis in the dermis, and the infiltration of inflammatory cells in the dermis [20]. To analyze the effects of bvPLA2 on DFE/DNCB-induced histopathological changes in the ear tissues, we assessed the epidermis, dermis thickness, and inflammatory index of skin lesions (Figure 3A,C–E). The AD group significantly increased the epidermis, dermis, and infiltration of inflammatory cells in the ear tissues compared to the NC group. Intriguingly, the ears of the bvPLA2-treated groups exhibited less epidermal and dermal hyperplasia compared to the AD group. Specifically, histological evaluation using H&E staining revealed that the epidermal hyperplasia and lymphocyte infiltration exerted by AD induction were attenuated by bvPLA2 treatment in a dose-dependent manner (16 and 80 ng/ear). These effects of bvPLA2 were not more significantly different than those of the DEXA group. We next performed toluidine blue staining to investigate the effect of bvPLA2 on DFE/DNCB-induced infiltration of mast cells and eosinophils into ear skin lesions. Mast cells act as the important effector cell in hypersensitivity through activation via the IgE receptor in AD [21]. As shown in Figure 3B,F, the accumulation of mast cells was significantly reduced in bvPLA2-treated groups. Taken together, these data indicate that bvPLA2 treatment exhibits a dose-dependent effect on the histopathological changes against DFE/DNCB-induced AD.

### 2.4. bvPLA2 Contributes to the Induction of Tregs in DFE/DNCB-Induced AD-Like Skin Lesions

We previously reported that bvPLA2 can induce Foxp3-expressing CD4^+^ regulatory T cells (Tregs) in OVA-induced asthma and acute kidney injury model [17]. Recent study has suggested that Tregs inhibit allergen-specific Th1 and Th2 cell responses, playing a role in the physiological immune response to allergen [22]. To confirm the involvement of bvPLA2 in the induction of Tregs to the skin lesions, the expression of Foxp3 in the ear tissue was determined by real-time PCR. As expected, bvPLA2 treatment showed significantly increased Foxp3 mRNA level in the AD-like lesions (Figure 3G).

### 2.5. Effects of bvPLA2 in DFE/DNCB-Induced AD-Like Skin Lesions through the Induction of Tregs

**Figure 4:**
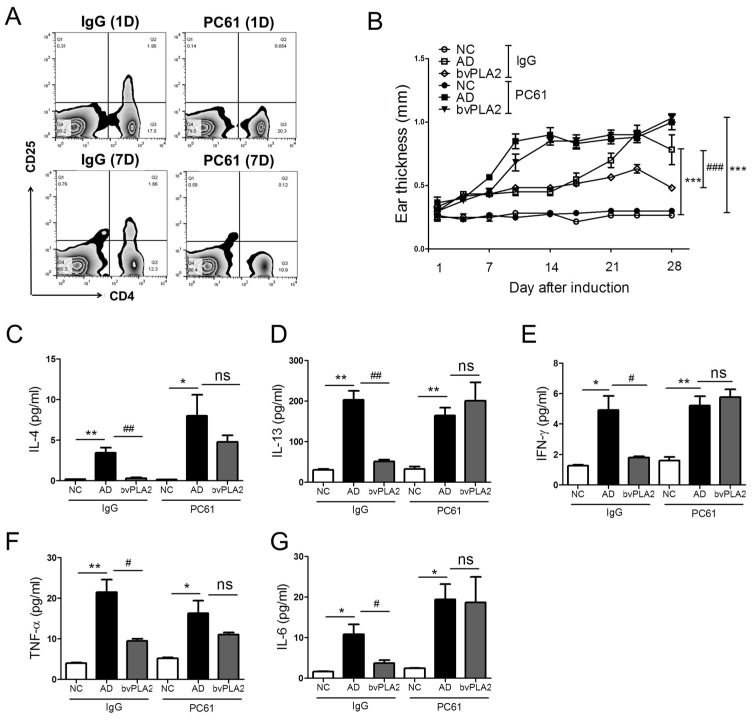
Effects of bvPLA2 on AD-like skin lesions of CD25 depleted mice. The female BALB/C mice received once with a dose of 0.25mg/kg of rat anti-mouse CD25 (PC61) or total rat IgG on days 3, 4, 11, 17, and 25. (**A**) CD4^+^CD25^+^ populations were measured using FACS on day 1 and 7; (**B**) The ear thickness was measured using a dial thickness gauge 24 h after the DFE/DNCB application. The concentration of (**C**) IL-4; (**D**) IL-13; (**E**) IFN-γ; (**F**) TNF-α; and (**G**) IL-6 were measured by mouse CBA (Th1, 2, and 17) kit and ELSA kit. NC: normal control group; AD: DFE/DNCB applied group; bvPLA2: DFE/DNC applied and bvPLA2 (80 ng/Ear)-treated group. IgG: non-CD25 depleted mice; PC61: anti-CD25-depleted mice. The statistical analyses were conducted with a one-way ANOVA followed by Newman-Keuls multiple comparisons test (** *p* < 0.01 and * *p* < 0.05, ^##^ *p* < 0.01 and ^#^ *p* < 0.05; not significant = ns, *n* = 3).

To investigate whether bvPLA2 has the potential to ameliorate AD-like skin lesions through the induction of Tregs, we depleted Tregs using an anti-CD25 mAb (clone: PC61) and determined CD4^+^CD25^+^ populations in spleen on day 1 and 7. Treatment of PC61 antibody completely eliminated CD25^+^ cells after one day of injection and the depletion was sustained for seven days (Figure 4A). The results showed that the bvPLA2 (isotype IgG) group significantly reduced ear thickness in the inflamed ear (Figure 4B). However, the Treg-depleted bvPLA2 (PC61) group did not show diminish ear thickness, but was also aggravated compared to the bvPLA2 (IgG) group. All cytokines were upregulated in the AD (IgG) group, and bvPLA2 suppressed the expression of all Th1 and Th2 cytokines. We found a consistent pattern of significant higher levels of Th1 and Th2 cytokines by bvPLA2 in Treg-depleted mice (Figure 4C–G). Altogether, these data indicated that although bvPLA2 can suppress the increase ear thickness and Th1 and Th2 cytokines expression in AD-like skin lesion mice, these effects were not observed following the Tregs depletion.

### 2.6. bvPLA2 Suppresses the Histopathological Changes via the Induction of Tregs

**Figure 5:**
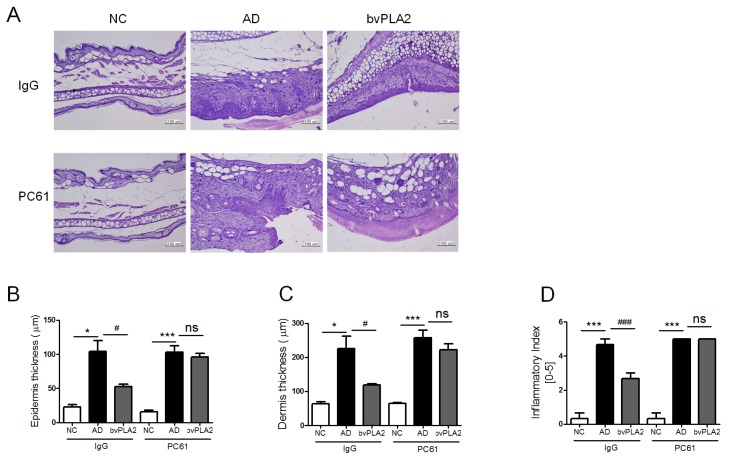
Effects of bvPLA2 on histological changes in AD-like skin lesions of CD25 depleted mice. (**A**) Representative ear section images were stained with H&E to assess the histological changes (×200 magnification); (**B**) the thicknesses of epidermis; and (**C**) dermis were quantified based on the H&E stained sections using Image Pro-Plus 5.1 software as described in the Materials and Methods; (**D**) Evaluation of ear histological change severity was quantified using a five-point score system. The statistical analyses were conducted with a one-way ANOVA followed by Newman-Keuls multiple comparisons test (*** *p* < 0.001 and * *p* < 0.05 and ^##^ *p* < 0.01 and ^#^ *p* < 0.05; not significant = ns, *n* = 3).

We also investigated the morphological changes of ear tissues in response to bvPLA2 and Treg depletion (Figure 5A). The bvPLA2 (IgG) group showed significant suppression of the elevated levels of epidermis and dermis thickness and infiltration of inflammatory cells in ear tissues. Although bvPLA2 can suppress the epidermal skin lesions in the DFE/DNCB-induced mice model, these effects were not observed in the Treg cell-depleted group. Taken together, these data demonstrated that bvPLA2 has potential to counteract AD-like skin lesion associated inflammation responses via the induction of Tregs.

## 3. Discussions

Atopic dermatitis (AD) is a chronic inflammatory skin disease, which is characterized by pruritus and eczematous skin lesions. The increased incidence of AD in children is closely related to a potential history of eczema and it has been suggested that a genetic background is important in the pathogenesis of AD [23]. The immunologic triggers differ among individuals and include various foods, airborne allergens, irritants and contactants, hormones, stress, climate, and microorganisms. AD is closely connected with other atopic diseases, most frequently as part of the atopic triad of childhood disease—allergic dermatitis, asthma, and allergic rhinitis [24]. In general, 50%–80% of AD patients develop asthma and/or allergic rhinitis.

In the present study, we adopted a mouse model of AD-like skin lesions inflammation through epicutaneous application of house dust mites, a common allergen associated with human AD. We clearly showed that typical AD symptoms were observed following the repeated application of DFE/DNCB. Ear swelling and fibrotic changes were detected, and the total IgE and Th1/Th2 cytokine levels dramatically increased. Histopathological analysis also revealed the infiltration of mast cells, which are important effector cells and a source of histamine into the skin lesions. bvPLA2 treatment significantly suppressed these AD symptoms, which suggests the beneficial effect of bvPLA2 on AD-like skin lesions.

AD skin lesions are characterized by the overexpression of the Th2 cytokines IL-4 and IL-13, and the Th22 cytokines IL-22. The overexpression of Th2 cytokines (IL-4, IL-5, and IL-13) leads to an immune response resulting in elevated serum IgE levels and eosinophil development [25]. Another group reported that the development of AD is associated with the overexpression of the Th2 and Th22 cytokines, whereas chronic inflammatory responses are mediated by a IFN-γ and IL-12-producing Th1 component and characterized by increased numbers of IgE-bearing cells in the epidermis and the domination of macrophages in the dermal mononuclear cell infiltration [26]. In our AD-like skin lesions model, the expression of Th1 and Th2 cytokines, and the serum level of IgE were markedly elevated. In addition, bvPLA2 suppressed both Th1 and Th2 responses both acute and chronic skin inflammation elicited by AD-like skin lesions.

Treg cells play critical roles in suppressing aberrant immune responses against self-antigens [27]. Decreased number of Tregs in inflammatory sites is related with inflammatory disease, including AD. Bae et al., reported that mice having dust mite-induced AD showed reduced Foxp3 expression in both inflamed site and dLN [28]. Loss of Tregs caused by Foxp3 deficiency caused a multi-organ inflammatory response including skin inflammation resembling AD associated with elevated serum IgE levels, eosinophilia, dysregulated Th1 and Th2 cytokine production, and other autoimmune symptoms [29,30]. The depletion of Treg cells during epidermal sensitization led to significantly exacerbated skin lesions, including increased expression of Th2 cytokines and serum IgE levels. These reports suggest that lack of Tregs might contribute to recapitulatation of important immunological features of AD. In the previous study, we reported that the protective role of bvPLA2 as a novel Foxp3^+^ Treg cell inducer in a mouse model of Parkinson’s disease [31]. Furthermore, we investigated that the involvement of Treg populations in the protective effects of bvPLA2 in DFE.DNCB-induced AD-like skin lesions. Depletion of Tregs using PC61 anti-CD25 mAb resulted in no significant reduction of epidermal thickness and Th1/2 cytokines such as IL-4, IL-13, IFN-γ, and IL-6 in bvPLA2-treated AD mice. Thus, bvPLA2 treatment ameliorates the skin lesions through the induction of Treg cells, which have an important role in controlling AD-like inflammation.

There is no known cure of AD patients, but skin lesions successfully treated by a variety of topical and/or systemic agents. These include prevention of Th2 responses, enhancement of Th1 responses and decrease of IgE concentration. Topical glucocorticosteroids are important and effective remedies for treatment of allergic skin diseases. However, it is well known that prolonged use of high doses of glucocorticoids causes a variety of adverse-effects. In this study, we examined the effects of bvPLA2 on AD-like skin lesions using the BALB/c mice model. Treatment with bvPLA2 significantly suppressed the increased levels of serum IgE and Th1 and Th2 cytokines. Histological changes and the infiltration of mast cells were attenuated by bvPLA2 application. The results of this study revealed that topical exposure to bvPLA2 aggravated atopic skin inflammation.

## 4. Materials and Methods

### 4.1. Animals

Female 7–8-week-old BALB/c mice were purchased from Charles River Korea (Orient Bio, Seungnam, Korea). Mice were housed under pathogen-free conditions with air conditioning on a 12-h light/dark cycle with free access to food and water during the experimental period (ad libitum). The study was conducted in accordance with the Rules for Animal Care and Guiding Principles for Experiment Using Animals and were approved by the University of Kyung Hee Animal Care and Use Committee (KHUASP(SE)-12-015, 15 July 2012).

### 4.2. Reagents

Bee venom phospholipase A2 (bvPLA2, *Apis mellifera*) and dexamethasone (DEXA) were purchased from Sigma (Sigma-Aldrich, St. Louis, MO, USA). The American house dust mite in the form of freeze-dried crude *Dermatophagoides farinae* extract (DFE) (Greer Laboratories, Lenoir, NC, USA) was used as an antigen and 2,4-dinitrochlorobenzene (DNCB) (Sigma-Aldrich) was used as a sensitizer to induce AD in the mice. bvPLA2 was dissolved in phosphate-buffered saline (PBS) and DFE was dissolved in PBS containing 0.5% Tween 20. DNCB was dissolved in acetone and olive oil (AOO) at a ratio of 3:1. AOO or AOO containing 0.5% Tween 20 was used as a vehicle.

### 4.3. Atopic Dermatitis Model

Induction of AD lesions was performed according to a previously described experimental protocol [32]. To induce AD lesions, mice were divided into five groups as follows (*n* = 5 per group). NC: normal control group; AD: DFE/DNCB applied group; DEXA: DFE/DNCB applied and dexamethasone-treated group (0.1 mg/Ear, 20 µL) as a positive control; bvPLA2 (16 ng/Ear): DFE/DNC applied and bvPLA2 (16 ng/Ear)-treated group; bvPLA2 (80 ng/Ear): DFE/DNC applied and bvPLA2 (80 ng/Ear)-treated group. The hair was removed with depilation lotion and surgical tape (Nichiban, Tokyo, Japan). After hair removal, 20 µL of 1% DNCB solution was applied to each ear and then 20 µL of 10mg/ml DFE was applied for sensitization four days later. The treatment of DFE or DNCB was repeated once a week for four weeks. For bvPLA2 treatment, mice were treated with bvPLA2 (16 and 80 ng/ear, approximately equivalent to 0.2 and 1 bee sting) applied with a brush four times per week for three weeks. Dexamethasone (50 µg/ear) was treated as positive control. On day 28, the ear thickness was measured using a dial thickness gauge (Kori Seiki MFG, Co., Tokyo, Japan) and mice were assessed with a dermatitis score via a blinded test and sacrificed. Blood samples were collected by orbital puncture and the ear tissues were removed for further analysis. The experiments were performed in duplicate.

### 4.4. Treg Depletion

Anti-mouse CD25 (clone: PC61) was produced in-house from hybridomas obtained from the American Type Culture Collection (ATCC; Manassas, VA, USA). Female BALB/C mice received one dose of 0.25 mg/kg of rat anti-mouse CD25 or rat IgG control (Sigma-Aldrich) on days 3, 4, 11, 17, and 25. The efficacy of the Treg depletion was analyzed on days 28 by FACSCalibur (BD Biosciences, San Diego, CA, USA) for CD4 and CD25 expression.

### 4.5. Evaluation of Skin Lesion

The severities of the DFE/DBCB-induced AD-like skin lesions were clinically evaluated using previously established methods [33,34]. The skin lesions on the ears were scored according to the following four symptoms: erythema/hemorrhage, edema, excoriation/erosion, and dryness/scaling. Dermatitis score was defined as the sum of the individual scores, which were graded follows: 0, no symptoms; 1, mild symptoms; 2, moderate symptoms; and 3, severe symptoms.

### 4.6. Determination of Serum IgE Titers

Blood samples were collected by centrifugation at 2500 *g* for 20 min. The serum was diluted (1:250) with 5% fetal bovine serum (FBS) in PBS (assay diluent) and the serum IgE levels were measured using a mouse IgE ELISA kit (BD Biosciences, San Diego, CA, USA) according to the manufacturer’s instructions. Briefly, a 96-well microtiter plate (Costar, NY, USA) was coated overnight at 4 °C with anti-mouse IgE mAb. After wash with PBS containing 0.05% Tween 20, the plates were blocked with 5% FBS in PBS for 1 h at RT. The diluted 100 µL serum samples were incubated for 2 h at RT. Secondary peroxidase-labeled biotinylated anti-rat IgE mAb was incubated in blocking buffer for 1 h. The enzyme reaction was initiated by addition of TMB substrate solution (BD Biosciences) for 30 min, and the reaction was stopped by the addition of 50 µL stop solution to each well. The optical density was measured at 450 nm with a microplate reader (SOFT max PRO, version 3.1. Molecular Devices, Sunnyvale, CA, USA). The lower limit of detection for the IgE ELISA was 1.5 ng/mL.

### 4.7. Assessment of Th1 and Th2 Cytokines in Ear Tissues Using ELISA

The levels of IL-4, TNF-α, IFN-γ, IL-6, and IL-13 in the ear tissues were determined using an ELISA kit (BD Biosciences and R&D, Minneapolis, MN, USA) according to the manufacturer’s protocols. Each ear tissue sample was prepared by homogenization in Tissue Protein Extraction Reagent (T-PER; Pierce Biotechnology Inc., Rockford, IL, USA) with 1 mg/ml protease inhibitor cocktail (Roche Diagnostics, Mannheim, Germany). 96-well microtiter plates were coated overnight at 4 °C with anti-mouse IL-4, TNF-α, IFN-γ, IL-6, or IL-13 mAbs in coating buffer. The plates were washed twice with PBS containing 0.05% Tween-20 and blocked with 5% FBS in PBS for 1 h at RT. After two washes, the plates were incubated for 2 h at RT with the ear tissue homogenates. The secondary peroxidase-labeled biotinylated anti-mouse IL-4, TNF-α, IFN-γ, IL-6, or IL-13 mAbs in assay diluents were incubated for 1 h. Finally, the plates were treated with TMB substrate solution for 30 min and the reaction was terminated by the addition of stop solution. The optical density was measured at 450 nm using a microplate reader (SOFT max PRO). The total protein concentrations were determined using a BCA kit (Pierce Biotechnology Inc.). The results were normalized to the total amount of ear tissue protein in each sample.

### 4.8. Histological Analysis

For the histopathological examinations, the ear lesioned skin of each mouse was removed and fixed with 10% neutral-buffered formalin for 24 h at 4 °C. The tissues were dehydrated, embedded in paraffin and cut into 4-μm-thick sections using a rotary microtome. The sections were stained with hematoxylin and eosin (H&E; Sigma-Aldrich) to evaluate the epidermal hyperplasia and the infiltration of the immune cells into the dermis. Toluidine blue (TB) staining was performed to measure the degree of mast cell infiltration. Images of the ear tissue sections were acquired using an Olympus BX51 microscope (Olympus, Tokyo, Japan) and quantified using Image Pro-Plus 5.1 software (Media Cybemetics Inc., Silver Spring, MD, USA).

### 4.9. Statistical Analysis

The statistical analysis of the data was conducted using Prism 5 software (GraphPad Software Inc., La Jolla, CA, USA, version 5.0). The data are presented as the means ± S.E.M. The differences between the study groups were determined using one-way ANOVA followed by Newman-Keuls multiple comparisons tests. *p* < 0.05 was considered statistically significant. The experiments with two groups were analyzed with unpaired two-tailed *t*-tests.
